# Recombineering strategies for developing next generation BAC transgenic tools for optogenetics and beyond

**DOI:** 10.3389/fnbeh.2014.00111

**Published:** 2014-04-03

**Authors:** Jonathan T. Ting, Guoping Feng

**Affiliations:** McGovern Institute for Brain Research and Department of Brain and Cognitive Sciences, Massachusetts Institute of TechnologyCambridge, MA, USA

**Keywords:** bacterial artificial chromosome, transgenic mice, BAC recombineering, Drd1a, Drd2, Adora2a, Chat, DAT

## Abstract

The development and application of diverse BAC transgenic rodent lines has enabled rapid progress for precise molecular targeting of genetically-defined cell types in the mammalian central nervous system. These transgenic tools have played a central role in the optogenetic revolution in neuroscience. Indeed, an overwhelming proportion of studies in this field have made use of BAC transgenic Cre driver lines to achieve targeted expression of optogenetic probes in the brain. In addition, several BAC transgenic mouse lines have been established for direct cell-type specific expression of Channelrhodopsin-2 (ChR2). While the benefits of these new tools largely outweigh any accompanying challenges, many available BAC transgenic lines may suffer from confounds due in part to increased gene dosage of one or more “extra” genes contained within the large BAC DNA sequences. Here we discuss this under-appreciated issue and propose strategies for developing the next generation of BAC transgenic lines that are devoid of extra genes. Furthermore, we provide evidence that these strategies are simple, reproducible, and do not disrupt the intended cell-type specific transgene expression patterns for several distinct BAC clones. These strategies may be widely implemented for improved BAC transgenesis across diverse disciplines.

## Introduction

Bacterial Artificial Chromosomes (BACs) are large DNA constructs composed of a small cloning vector backbone ligated to large fragments of restriction-digested genomic DNA that can be stably propagated as well as manipulated in bacterial host cells. Extensive BAC libraries have been constructed with genomic material from a variety of organisms and have served as indispensable tools for large-scale genome sequencing and mapping efforts. One such project culminated in the release of three landmark mouse BAC libraries derived from the C57BL/6J and 129S6/SvEvTac strains with a combined 30-fold coverage of the mouse genome (Osoegawa et al., 2000). This resource was quickly tapped to create the first BAC transgenic mouse line with functional transgene expression driven by successful integration of an engineered Bacterial Artificial Chromosome into the mouse genome (Yang et al., 1997). This pioneering work established the feasibility of diverse targeted manipulations of BAC clones in *E. coli* by homologous recombination, a method now commonly referred to as BAC recombineering (recombination-mediated genetic engineering). Furthermore, this work boldly asserted the incredible potential of BACs for gene therapy, disease modeling, and other basic research applications aimed at deciphering gene function.

The ability to obtain and manipulate BAC clones with genomic DNA spanning hundreds of kilobases is highly advantageous for transgenic applications given that most mouse BAC clones (average insert size 150–200 kb) encompass one or more genes including flanking regions essential to instructing cell-type specific expression patterns *in vivo*. Furthermore, the presence of large spans of insulating genetic material around a transgene expression cassette can curb transgene silencing or mosaic expression *in vivo* due to position effect variegation following random integration into unfavorable sites of the host genome (Bian and Belmont, 2010). Indeed, Yang et al. (1997) proposed that BAC transgenesis would be especially expedient for creating mice with faithful cell-type specific expression of Cre recombinase for gene disruption in mice, an idea that directly catalyzed the immensely successful Gene Expression and Nervous System ATlas (GENSAT) project at Rockefeller University led by Nathaniel Heintz and colleagues (Gong et al., 2003; Heintz, 2004). Importantly, BAC transgenic Cre driver rodents (particularly the extensive collection from the GENSAT project) have played a pivotal role in expanding the utility of emerging optogenetics-based technologies in neuroscience by enabling unprecedented access to monitor and manipulate genetically-defined cell populations in the nervous system when used in combination with Cre-inducible expression strategies for diverse optogenetic probes (Atasoy et al., 2008; Cardin et al., 2009; Petreanu et al., 2009; Witten et al., 2011; Madisen et al., 2012; Saunders et al., 2012; Zariwala et al., 2012). In addition, numerous laboratories have tapped into the knowledge base of the GENSAT project to guide the development of new tools such as the first collections of BAC transgenic mice with direct cell-type specific expression of ChR2 in the central nervous system (Hagglund et al., 2010; Zhao et al., 2011). Collectively, these BAC transgenic tools are now widely implemented for optogenetic deconstruction of complex neural circuits that mediate diverse animal behaviors and brain states (Tsai et al., 2009; Kravitz et al., 2010; Witten et al., 2010; Aponte et al., 2011; Halassa et al., 2011; Yizhar et al., 2011; Tai et al., 2012; Tan et al., 2012; Bock et al., 2013; Chaudhury et al., 2013; Cui et al., 2013; Stamatakis et al., 2013; Steinberg et al., 2013). The broader topic of optogenetic dissection of behavior in mammalian model systems has been extensively reviewed elsewhere (Tye and Deisseroth, 2012; Yizhar, 2012; Lenz and Lobo, 2013; Nieh et al., 2013).

Here we propose strategies for developing the next generation of BAC transgenic lines that are devoid of overexpressed extra genes. We describe multiple BAC recombineering strategies for eliminating undesirable extra genes from BAC clones in order to circumvent confounds due to overexpression of such extra genes in BAC transgenic lines. In addition, we demonstrate that these modification procedures may be performed in parallel or sequentially with routine BAC recombineering steps for introducing a transgene of interest under the control of cell-type specific promoter elements. Together, these procedures effectively result in pure transgenic expression cassettes ≥65 kb in size that can be used in pronuclear injection to produce BAC transgenic animals. These steps are simple, efficient, reproducible, and can be implemented for the modification of any BAC clone. We are applying these methods in developing the next generation of BAC transgenic animals for optogenetics-based research and expect these strategies may be widely adapted across diverse disciplines.

## Materials and methods

### Targeting vector construction

A pBlueScript-derived vector iTV1 was used to generate BAC targeting vectors. iTV1 contains cloning sites for A and B homology arms which flank a large multiple cloning site, bovine growth hormone polyadenylation (BGHpA) signal, and FRT flanked neomycin resistance cassette (FRT-NEO-FRT). First, iTV1 was modified by addition of the woodchuck post-translational regulatory element (WPRE) upstream of the BGHpA to create iTV1-WPRE. This modification was chosen to improve transgene protein expression level and RNA stability. BAC specific homology arms A and B (400–600 bp each arm) were PCR amplified from BAC template DNA and cloned into iTV1-WPRE. In a separate mammalian expression vector, two copies of ChR2(E123T/H134R), herein referred to simply as ChETA_TR_, and the orange/red emitting tdTomato fluorophore were combined using viral 2A linkers. We selected P2A with a C-terminal GSG linker because this combination was shown to be the most efficiently processed of all viral 2A elements (Kim et al., 2011) and thus was the most likely to enable a complete physical uncoupling of the opsin and fluorophore with no significant unprocessed protein fraction. The ChETA_TR_-P2A-ChETA_TR_-P2A-tdTomato cassette (i.e., 2xChETA-P2A-tdTomato) was then sub-cloned into the large multiple cloning site of iTV1-A/B-WPRE to complete the BAC targeting vectors. In this final cloning step great care was taken to ensure minimal disruptions to the junction between the A Box and the start of the ChETA sequence, except for addition of a Kozak consensus sequence.

### BAC trimming cassettes

The pBlueScript-based deletion cassettes were constructed by standard PCR and cloning methods. The order of assembled features is as follows: (1) a 50–350 bp BAC-specific homology arm (selected to target the preferred location for BAC trimming in a BAC clone of interest), (2) AscI and NotI restriction sites (for later removal of the BAC vector during purification of BAC DNA for pronuclear injection), (3) the *bla* gene encoding ampicillin resistance (AmpR), and (4) a “loxP deletion” homology arm (targeting the BAC vector sequence adjacent to the wild-type loxP site in pBACe3.6 or pTARBAC1 vectors).

loxP deletion arm: 5′-gttaacgtgccggcacggcctgggtaaccaggtattttgtccacataaccgtgcgcaaaatgttgtggataagcaggacacagcagcaatccacagcaggcatacaaccgcacaccgaggttactccgttctacaggttacgacgacatgtcaatacttgcccttgacaggcattgatggaatcgtagtctcacgctgatagtctgatcgacaatacaagtg-3′

BAC clone specific deletion arms:

Adora2a BAC (RP24-238K3): 5′-gagctgagtggccagcgacctattgcctaggcatagataaccatatatca-3′

Drd2 BAC (RP23-161H15): 5′-gagaccagtgccagcagaagctatggtcattgtggtgataggagcgtggctga-3′

Chat BAC (RP24-256F2): 5′-ttcagtccactatactttcctgcttttcttcatggcttagcaaggttcctggacctcagcagagttaatagaaaatgcaggctgcaactggatggttagcgatgaaactaagcaactctagacagtgcagtcagacacatactttctctaactggcgggagttactcactcacgcaatcacctctaacacttaaccacagcaggtggaaattgagttagtttaagaggctaactctgtgctaagcctggggacttgggacaggaaagccttggccccgcccagcagtggccccgcccacctctctgaaggctggactggcggttgcctagcagcagg-3′

DAT BAC (RP24-269I17): 5′-agatataacctacctttgcatgttagccaggattaagatttatattaccg-3′

Pvalb BAC (RP24-306A6): 5′-ttccggaaggtgcacagcacgggtgctgtccgcagttgtttgctgtgcaagcatgcagggccttcctgtcttcctcactgctcaacgttgcacatttttccctccccgctatttcagtttttagggttcataacatgcttgggctttaaagatggcatttcgattctggacgtgaa-3′

Vgat BAC (RP24-246L1): 5′-agttccatccctggaacctattgggtgaagagaacaggcatcttggcacgctcccacacccatcacgcaataaataaacaaatgaacagaaaaagaagtcaaaggggaaggagattagcttaacacggtcacaaaactaagggctagatctgcaaggctatgcagacagagaccaagggagagtaaggggacagggaggggcggagtccagcccagtgtgggtggagcctttggtcccttagttagagca-3′

### Gene inactivation cassettes

Sfxn1 homology arms A and B (1 kb each) were PCR amplified and cloned into pBlueScript II. A 29 bp dsOligo containing a three-frame translational stop mutation and diagnostic SalI restriction site (5′-gaattc**tag**a**taa**c**tag***gtcgac*ctgcag-3′), was synthesized and cloned between the two homology arms. This vector is designed to insert the three-frame translational stop cassette after the 13th codon in the Sfxn1 coding region in the *Drd1a*-spanning BAC clone RP23-47M2 and was thus called “Sfxn1 Target.” A second vector “VAChT Target” was designed with the identical strategy except that homology arms A and B directed the insertion of the three-frame translational stop cassette after the 5th codon in the *Slc18a3* coding region (VAChT locus) in the *Chat*-spanning BAC clone RP24-256F2. Relatively long 1 kb homology arms were selected in order to ensure a high efficiency of homologous recombination since this targeting cassette does not contain a selection marker for screening and must be used in combination with a separate iTV1-based targeting vector.

Critical regions of all constructs were verified by DNA sequencing. The targeting vectors were linearized by restriction digestion and purified in preparation for subsequent use in BAC recombineering steps. Care was taken to ensure adequate duration of restriction digestion (or in some cases two sequential rounds of digestions were performed) such that there was no contaminating uncut plasmid carried over into the electroporation step.

Additional information including complete DNA sequences of the targeting vectors and modified BAC clones used in this study are available upon request.

### BAC recombineering

Appropriate BAC clones were selected from the RP23/RP24 C57 mouse BAC library following a detailed analysis using the Esembl Genome Browser (http://www.Ensembl.org) and were obtained from the Children's Hospital Oakland Research Institute (CHORI). The intact BAC DNA was isolated from the original host DH10B strain by BAC mini-prep and verified by restriction digestion and pulsed field gel electrophoresis (PFGE). Successfully verified BAC DNA was transferred into the EL250 strain in preparation for recombineering steps and maintained as glycerol stocks stored at −80°C.

Most often the first strategic recombineering step was BAC trimming to delete unwanted regions of BAC DNA harboring extra genes. 20 mL of LB media plus chloramphenicol were inoculated with EL250 cells containing the target BAC clone and placed in a shaking incubator at 32°C for 2 h until the cells reached an early log growth phase. The culture was induced for 15 min at 42°C in a shaking water bath to permit transient expression of genes required for homologous recombination. Cells were rapidly chilled, pelleted, and washed three times with ice-cold MilliQ water to remove salts. Approximately 10–100 ng of linearized BAC trimming vector was added to 50 μL of induced cells in a 1 mm gap cuvette and the mixture was immediately electroporated (1.75 kV, 25 μF, 200 Ohms). The cells were recovered and plated onto LB plus chloramphenicol and carbenicillin to select for successful homologous recombination events, meaning insertion of the AmpR cassette in place of the targeted region. Double resistant clones were picked for further analysis to verify the deletions.

In a second round of targeting the virtually identical steps were performed to insert the *ChETA*_TR_ transgene expression cassette at the initiating methionine start codon of the targeted gene within the selected BAC clone by homologous recombination. In this case, successfully modified clones were resistant to chloramphenicol, carbenicillin, and kanamycin due to insertion of the FRT-NEO-FRT in the targeting vector. (Note: although it is easily feasible to perform the first and second targeting events in a single step due to the insertion of two different selection cassettes, it was preferable to do these steps in series in order to save a glycerol stock of the successfully trimmed BAC in EL250 cells for future reuse). The NEO cassette was then excised from the modified BAC by arabinose induction of flp recombinase in the EL250 cells. Modified BAC clones were extensively screened for accuracy and the correctly targeted BAC DNA was grown in large scale and purified using the BAC100 kit (Clontech). 10–15 μg of BAC DNA was restriction digested with NotI or AscI to liberate the BAC vector from insert and the linear fragments were separated by PFGE. The intact BAC insert band was excised from the PFG, electroeluted, and spot dialyzed against fresh microinjection buffer.

For gene inactivation recombineering experiments the linearized Sfxn1 Target DNA was combined at ~20-fold molar excess with linearized iTV1-D1-ChETA targeting construct DNA immediately prior to the electroporation step. This was best accomplished by diluting linearized iTV1-D1-ChETA targeting DNA 1:20 in water and then adding approximately equal volumes of linearized Sfxn1 Target and diluted iTV1-D1-ChETA into the cuvette for the electroporation into induced EL250 cells propagating the *Drd1a*-spanning BAC RP23-47M2. Double recombinants were identified by resistance to chloramphenicol and kanamycin and further verified by PCR screening and subsequent restriction digestion and PFGE of isolated BAC DNA to test for the incorporation of the diagnostic *SalI* site. Large scale BAC DNA isolation and processing for pronuclear injection were carried out as indicated above. In additional experiments we used VAChT Target to inactivate *Slc18a3* in the Chat-spanning BAC RP24-256F2. This dual targeting step was carried out together with the iTV1-Chat-ChETA targeting vector following identical methods as for Sfxn1 inactivation.

### Pronuclear injection and identification of transgenic founders

Transgenic mice (pure C57BL/6 Taconic) were generated by pronuclear injection of the highly purified intact BAC DNA into fertilized oocytes at a concentration of 0.5–2.0 ng/μL. Genotypes were determined by PCR from mouse tail DNA samples and line-specific primer sets. Mice that had the transgene integrated in the genome were kept as founders to establish distinct lines by mating to C57BL/6J mice. One line each of *Slc6a3-2xChETA_TR_-P2A-tdTomato* (DAT-ChETA), *Drd1a-2xChETA_TR_-P2A-tdTomato* (D1-ChETA), and *Adora2a-2xChETA_TR_-P2A-tdTomato* (A2A-ChETA) were established for further analysis. Only hemizygous mice on a pure C57 background were used for experiments in this study. All research involving mice was conducted according to the Institutional Animal Care and Use Committee guidelines at MIT. All procedures were approved by the Institutional Animal Care and Use Committee at MIT.

### Slice immunostaining and Confocal imaging

Mice were anesthetized with tribromoethanol (Avertin) and transcardially perfused with phosphate buffered saline (137 mM NaCl, 10 mM NaH_2_PO_4_, and 2.7 mM KCl, pH = 7.4; PBS) followed by 10% neutral-buffered formalin (Sigma-Aldrich, St Louis, MO). Brains were post-fixed in 10% formalin at 4°C overnight. Fixed brains were sectioned (50 μm) on a vibratome (Leica Microsystems, Buffalo Grove, IL) and the free-floating sections were washed for 10 min three times with PBS. Sections were then blocked for 1 h at room temperature in PBS containing 5% normal goat serum, 2% bovine serum albumin and 0.2% Triton-X 100. After blocking, the sections were incubated overnight at 4°C in blocking buffer containing one or more of the following antibodies: anti-ChAT (1:200 dilution; Millipore AB144P), anti-2A (1:500 dilution; Millipore ABS31), anti-red fluorescent protein (1:500 dilution; Rockland 600-401-379), anti-GFP (1:5000 dilution; Abcam AB6566), anti-DAT (1:300 dilution; Millipore MAB369), or anti-DARPP-32 (1:2000 dilution; BD Transduction 611520). Following the overnight incubation, sections were washed for 20 min three times with PBS and then incubated for 1 h at room temperature in Alexa dye-conjugated secondary antibody (1:1500; Invitrogen, Grand Island, NY). Alexa dyes utilized were: Alexa Fluor 488 (A-10667, goat anti-mouse and A-11034, goat anti-rabbit), and Alexa Fluor 555 (A-21429, goat anti-rabbit). Sections were washed for 20 min three times with PBS, dried on a glass microscope slide and mounted with VectaShield (Vector Laboratories, Burlingame, CA). Slides were stored at −20°C and were thawed to room temperature immediately prior to imaging. Whole brain montage images were acquired with CellSens software on an Olympus BX61 equipped with a motorized stage and epifluorescence illumination. Confocal z-stack images were acquired on an Olympus Fluoview 1000 laser scanning confocal microscope equipped with 488 and 543 nm laser lines.

## Results

### Implementing well characterized BAC clones to achieve reproducible cell-type specific transgene expression

**Figure 1:**
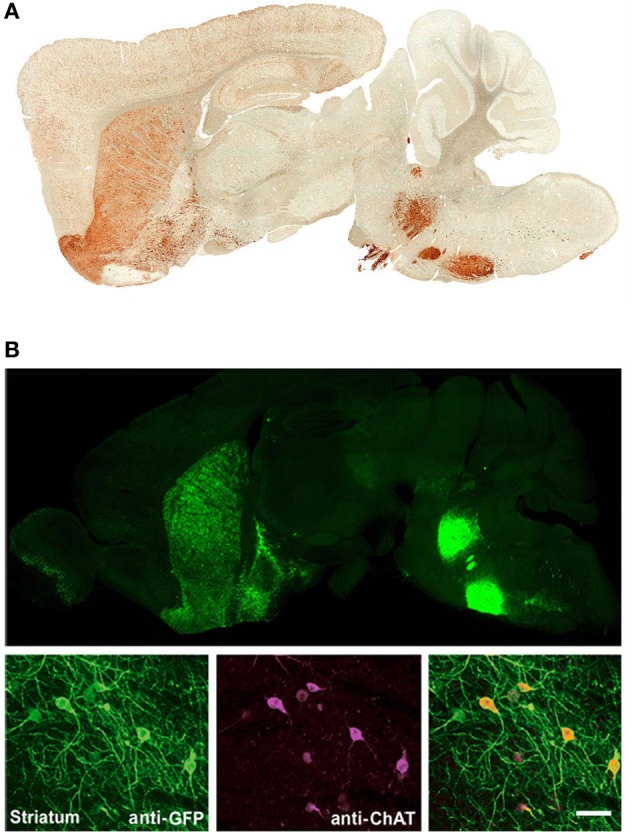
**Independent BAC transgenic mouse lines created by modification of the same original BAC clone exhibit highly reproducible cell-type specific transgene expression that recapitulates endogenous gene expression patterns. (A)** Sagittal brain section showing EYFP expression for *Chat-EYFP* line GH293 mice from the GENSAT project. **(B)** *Upper*, sagittal brain section showing native EYFP fluorescence for *Chat-ChR2(H134R)-EYFP* line 6. *Lower*, high magnification images of anti-GFP and anti-ChAT co-immunostaining demonstrating excellent co-localization. **(B)** Adapted from Zhao et al. (2011). Scale bar: 100 microns. Note the virtually identical pattern of transgene expression between these two independent lines shown in **(A,B)** with strong signal detected in known cholinergic nuclei including brainstem motor nuclui (facial nucleus and motor trigeminal nucleus), striatum, olfactory tubercle, and basal forebrain. Both lines were created by modification of BAC clone RP23-246B12 to insert the transgene of interest in place of the *Chat* gene.

We have previously developed several BAC transgenic mouse lines for expression of ChR2 in diverse neuronal subsets of the nervous system (Zhao et al., 2011). Our general strategy has been to select BAC clones that have previously been validated in the GENSAT project, as determined by the successful development of mouse lines for cell type-specific expression of Cre or EGFP. With this strategy we have observed that independent BAC transgenic mouse lines created by modification of the same original BAC clone exhibit highly reproducible cell-type specific transgene expression that recapitulates endogenous gene expression patterns. This is exemplified in the case of the *Chat*-spanning BAC clone RP23-246B12, which was successfully used to express both EGFP and ChR2-EYFP in cholinergic neurons of the nervous system (Figure 1). Thus, a known BAC clone can be modified for expression of virtually any transgene of interest in a well-defined neuronal subset with a relatively high probability of success following pronuclear injection.

### Most BAC clones used to make BAC transgenic lines span multiple genes

**Figure 2:**
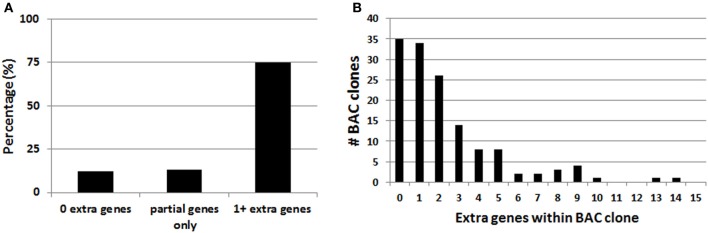
**Analysis of extra genes spanned by BAC clones of the GENSAT Cre driver repository. (A)** Plot of the percentage of BAC clones with either zero, partial gene fragments only, or 1 or more extra genes spanned. **(B)** Plot of the full distribution of extra gene number for the 139 BAC clones analyzed. Only intact genes that were fully contained within the BAC clone were counted.

In the course of this work it was noted that the majority of BAC clones selected for making BAC transgenic mice and rats contained multiple extra genes in addition to the explicitly targeted region (Table 1). Presumably this relates to the conventional wisdom that larger BAC clones spanning the targeted region are more likely to encompass the necessary regulatory elements for recapitulating the desired transgene expression pattern. Thus, in order to more broadly assess this issue of increased gene dosage for off-target genes we analyzed the entire collection of BAC clones from the GENSAT Cre driver mouse repository (http://www.gensat.org/cre.jsp). In total, 139 BAC clones from the RP23/RP24 C57 library were located and viewed with alignment to the mouse genome using the Ensembl Genome Browser (http://www.ensembl.org). This enabled us to determine the number of extra genes (i.e., genes other than the targeted gene region) spanned by each unique BAC clone. We report that 75% of the BAC clones used to derive GENSAT Cre driver lines contain at least one or more extra gene (Figure 2A), and in the extreme cases up to 14 independent and complete extra genes were spanned by a single BAC clone (e.g., RP23-368D24 used to create *Thbs*-Cre lines SW48-Cre and SW52-Cre; Figure 2B). Only 25% of BAC clones were entirely devoid of extra genes (12%) or only contained incomplete portions of gene coding regions (13%). Notably, many of the analyzed BAC clones used to derive GENSAT Cre driver lines were also previously implemented for making EGFP reporter lines.

**Table 1:** **BAC transgenic lines typically have increased gene dosage of one or more extra gene**.

**Name**	**Species**	**mouse BAC ID**	**Targeted gene**	**Extra genes in BAC clone**	**Citations**	**Unique lines**
*Adora2a-Cre*	mouse	RP24-238K3	*Adora2a*	Cytsa^*^, 1110038D17Rik-210^*^, Upb1	Gong et al., 2007	KG139
*Adora2a-EGFP*	mouse	RP24-238K3	*Adora2a*	Cytsa^*^, 1110038D17Rik-210^*^, Upb1	Gong et al., 2003	EP141
*Adora2a-hM3D*	mouse	RP24-238K3	*Adora2a*	Cytsa^*^, 1110038D17Rik-210^*^, Upb1	Farrell et al., 2013	AD6
*Chat-ChR2*	mouse	RP23-246B12	*Chat*	*Ogdhl, 1700024G13Rik, Slc18a3, Ercc6^^^*	Zhao et al., 2011	Line 5, Line 6
*Chat-Cre*	mouse	RP23-246B12	*Chat*	*Ogdhl, 1700024G13Rik, Slc18a3, Ercc6^^^*	Gong et al., 2007	GM24, GM60, GM53
*Chat-Cre*	rat	RP23-246B12	*Chat*	*Ogdhl, 1700024G13Rik, Slc18a3, Ercc6^^^*	Witten et al., 2011	Line 5
*Chat-EGFP*	mouse	RP23-246B12	*Chat*	*Ogdhl, 1700024G13Rik, Slc18a3, Ercc6^^^*	Gong et al., 2003	GH293
*Chat-EGFP*	mouse	RP23-268L19	*Chat*	*1700024G13Rik, Slc18a3, Ercc6^^^*	Tallini et al., 2006	Line *2*
*Chrm4-EGFP*	mouse	RP23-138P5	*Chrm4*	Ambra1^*^, Gm9821, Mdk, Dgkz, Creb3l1^*^	Lobo et al., 2006	Y86
*Drd1a-Cre*	mouse	RP23-47M2	*Drd1a*	*Sfxn1*	Gong et al., 2007	FK150, EY262, EY217
*Drd1a-EGFP*	mouse	RP23-47M2	*Drd1a*	*Sfxn1*	Gong et al., 2003	X60
*Drd1a-tdTomato*	mouse	RP23-47M2	*Drd1a*	*Sfxn1*	Ade et al., 2011	Line 6
*Drd2-Cre*	mouse	RP23-161H15	*Drd2*	*Ttc12, Ankk1?*	Gong et al., 2007	ER43, ER44
*Drd2-EGFP*	mouse	RP23-161H15	*Drd2*	*Ttc12*	Gong et al., 2003	S118
*Pvalb-ChR2*	mouse	RP23-305H12	*Pvalb*	*Ift27, Cacng2*	Zhao et al., 2011	Line 15
*TH-Cre*	mouse	RP23-350E13	*TH*	*GM6471, Ascl2*	Gong et al., 2007	FI12, FI172
*TH-Cre*	rat	RP23-350E13	*TH*	*GM6471, Ascl2*	Witten et al., 2011	Line 3
*Tph2-ChR2*	mouse	RP23-112F24	*Tph2*	Tbc1d15^*^	Zhao et al., 2011	Line 5
*VGAT-ChR2*	mouse	RP23-392P11	*Viaat (VGAT)*	Arhgap40, Ralgapb^*^, Adig, Actr5, Ppp1r16b^^^	Zhao et al., 2011	Line 8
*Viaat-Cre*	mouse	RP23-392P11	*Viaat (VGAT)*	Arhgap40, Ralgapb^*^, Adig, Actr5, Ppp1r16b^^^	Hagglund et al., 2013	–
*Viaat-Cre*	mouse	RP23-392P11	*Viaat (VGAT)*	none reported	Chao et al., 2010	Tg2.1

* *Partial gene that is missing the first coding exon containing ATG*.

^ *Truncated gene*.

### Extra genes contained within BAC clones can result in unintended gene overexpression in BAC transgenic lines

**Figure 3:**
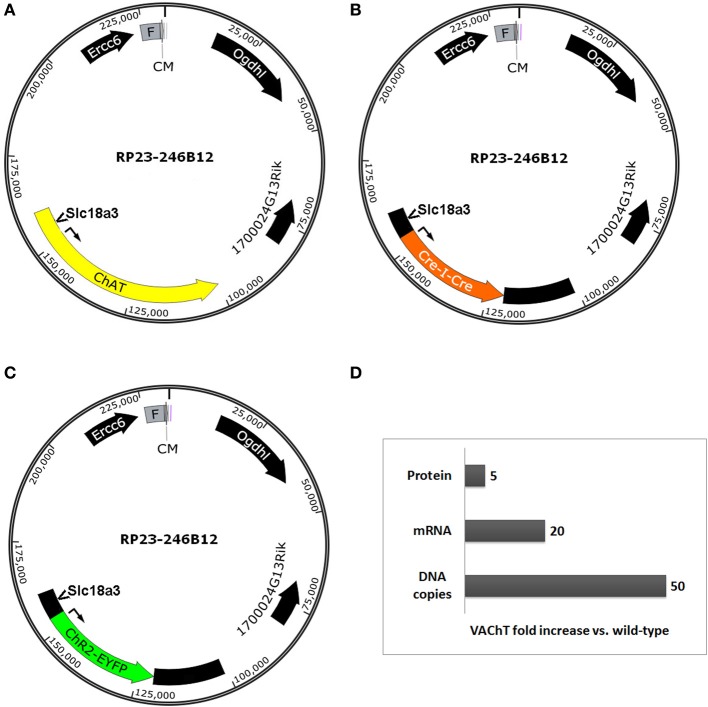
**Extra genes contained within BAC clones can result in unintended gene overexpression in BAC transgenic lines**. Simplified diagrams of the *Chat*-spanning BAC clone RP23-246B12 **(A)** that was modified for expression of **(B)** Cre recombinase (GENSAT BAC ID BX1191) or **(C)** *ChR2(H134R)-EYFP* under the control of the *Chat* gene promoter elements. Several extra genes are contained in the BAC clone, including *Ercc6* (partial), *Ogdhl, 1700024G13Rik*, and *Slc18a3*. Notably, *Slc18a3* (the gene encoding VAChT) is nested within the first intron of the *Chat* gene. **(D)** Summary of fold changes in VAChT levels (DNA copies, mRNA, and protein) in brain tissue from *Chat-ChR2(H134R)-EYFP* line 6 BAC transgenic mice relative to non-transgenic controls. This chart is based on data presented in Kolisnyk et al. (2013). Abbreviations: F, F-factor replicon; CM, chloramphenicol resistance gene. Both elements are part of the BAC vector.

The infrequently discussed down side of selecting very large BAC clones for BAC transgenesis is the potential for overexpression of unwanted extra genes and any associated behavioral, biochemical, or electrophysiological confounds. We previously developed a ChAT-ChR2-EYFP BAC transgenic mouse line (line 6) that enables optogenetic control of cholinergic neuron firing *in vitro* and *in vivo* (Ren et al., 2011; Zhao et al., 2011; Ma and Luo, 2012). The BAC clone that was used to create our ChR2-EYFP line was the same as the clone used to create independent ChAT-Cre mouse and rat lines (Gong et al., 2007; Witten et al., 2011). This *Chat*-spanning BAC clone contains the additional genes *Ercc6* (partial), *Ogdhl, 1700024G13Rik* and *Slc18a3* (Figures 3A–C). Importantly, the *Slc18a3* gene is nested within the first intron of the *Chat* gene and encodes for the vesicular acetylcholine transporter (VAChT), the overexpression of which has profound implications for cholinergic function. This prediction was confirmed in a recent study that provided direct evidence for increased VAChT expression in ChAT-ChR2-EYFP line 6 mice, and linked this VAChT overexpression to increased cholinergic tone, enhanced motor endurance, and attention and memory deficits (Kolisnyk et al., 2013). More specifically, the authors reported a 20-fold increase in VAChT transcript level in striatum and a 5-fold increase in VAChT protein level in hippocampus with no significant elevation of ChAT expression in either region (summarized in Figure 3D). The protein overexpression was associated with a 3-fold increase in stimulus-evoked ACh release in hippocampal slices, consistent with the behavioral data that VAChT overexpression has important functional consequences (Kolisnyk et al., 2013). Furthermore, striatal VAChT overexpression and elevated ACh release are associated with increased severity of psychomotor stimulant-induced repetitive behaviors in ChAT-ChR2-EYFP line 6 BAC transgenic mice (Jill R. Crittenden and Ann Graybiel, pers. commun.).

### Strategy for deletion of extra genes by BAC trimming

**Figure 4:**
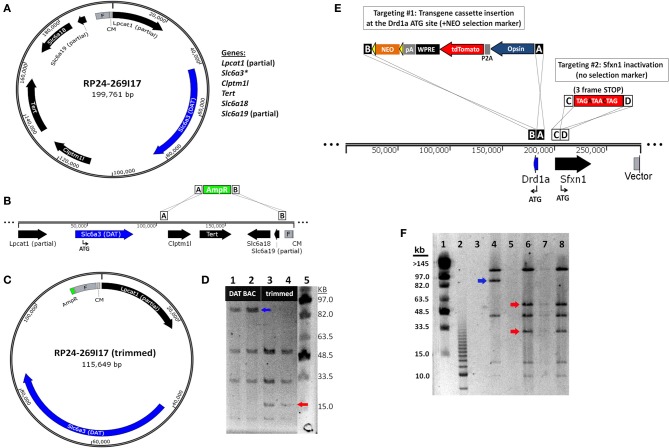
**BAC recombineering strategies for avoiding expression of extra genes. (A)** Strategy for deletion of extra genes by BAC trimming. A diagram of the *DAT* (*Slc6a3*)-spanning BAC clone RP24-269I17 reveals the presence of many extra genes including *Clptm1l*, *Tert*, *Slc6a18* and the truncated genes *Lpcat1* and *Slc6a3*. **(B)** The extra genes *Clptm1l*, *Tert*, *Slc6a18*, and *Slc6a19* located in the 3' flanking region of the *DAT* gene are deleted by homologous recombination. The recombination event replaces the ~84 kb BAC region with a 1 kb ampicillin resistance (AmpR) cassette. **(C)** Diagram of the “trimmed” *DAT* BAC clone that can now be used for insertion of a transgene cassette in place of the *DAT* gene by BAC recombineering. **(D)** Verification of DAT BAC trimming by diagnostic restriction digestion with SalI and PFGE. Lane 1 and 2: modified DAT BAC DNA. Lane 3 and 4: trimmed DAT BAC DNA. Lane 5: MidRange I PFG Marker. In successfully trimmed BAC clones the 91 kb DNA fragment (blue arrow) is reduced to 17 kb (red arrow). In addition, a 6 and 4 kb fragment are deleted but are too small to be visualized on the PFG. **(E)** Strategy for inactivation of extra genes by dual targeting. The *Drd1a*-spanning BAC clone RP23-47M2 contains the extra gene *Sfxn1* in the promoter region of the *Drd1a* gene. In the dual targeting strategy, two simultaneous homologous recombination events must occur. The first targeting event is the insertion of the transgene cassette (including the NEO selection marker) in place of the *Drd1a* gene. The second targeting event is the insertion of a triple frame stop mutation (and SalI restriction site) immediately downstream of the *Sfxn1* start codon. Double recombinant clones are identified by kanamycin resistance. **(F)** Verification of *Sfxn1* gene inactivation in modified *Drd1a* BAC DNA by diagnostic restriction digestion with SalI and PFGE. Lane 1: MidRange I PFG Marker. Lane 2: 2.5 kb marker. Lane 4: *Drd1a* BAC DNA (+*Sfxn1*). Lanes 6–8: *Drd1a* BAC DNA following *Sfxn1* inactivation. For successfully modified BAC clones the ~95 kb DNA fragment (blue arrow) is cleaved into 60 and 35 kb fragments (red arrows). Note that all other bands remain unaltered.

The most straightforward approach for eliminating extra genes contained in BAC clones is to remove segments from either end of the BAC insert, a method known as BAC trimming or shaving. We implemented a versatile method for BAC trimming in which small antibiotic selection cassettes are used to replace precise BAC DNA segments using a recombineering step in *E. coli* (Hill et al., 2000; Testa et al., 2003, 2004). The selection cassettes were PCR amplified using long primers to add ~50 bp homology arms to either end, and then used for recombineering in EL250 cells. The homology arms define the exact BAC region that will be deleted by the insertion of the selection cassette, and the selection cassette enables subsequent identification of properly modified BAC clones. In addition, many different selection cassettes can be utilized for BAC trimming (e.g., ampicillin resistance-*bla*, kanamycin resistance-*neo*, chloramphenicol resistance-*cam*, blasticidin resistance-*bsd*, and streptomycin resistance-*rpsL*), providing options for sequential rounds of BAC trimming and transgene targeting using different selection markers. To further generalize this strategy for deleting fragments from any BAC clone in the RP23 and RP24 library, in subsequent experiments one of the homology arms was designed on the end of the BAC vector such that only the second homology arm needs to be customized for each unique BAC clone to define the deletion region—the region containing undesirable extra genes. In addition, we added NotI and AscI restriction sites adjacent to the unique homology arm but before the selection cassette. Once the deletion of the BAC segments was confirmed by restriction digestion and pulsed field gel electrophoresis (PFGE) the BAC insert was liberated from the BAC vector and selection cassette by NotI or AscI digestion and subjected to further purification. We have used this method to successfully delete precisely defined BAC DNA segments, as demonstrated for the *Slc6a3*-spanning BAC clone RP24-269I17 in which the extra genes *Clptm1l*, *Tert*, *Slc6a18*, and *Slc6a19* located in the 3′ flanking region of the *Slc6a3* (DAT) gene were deleted by homologous recombination in which an ~84 kb BAC region was replaced with a 1 kb ampicillin resistance (AmpR) cassette (Figures 4A–C). The “trimmed” BAC clone can then be used for insertion of a transgene cassette in place of the *Slc6a3* gene by standard BAC recombineering using an iTV-based targeting vector (see Materials and Methods). The successful DAT BAC trimming was verified by diagnostic restriction digestion with SalI and PFGE (Figure 4D).

It is worth noting that other methods have been described for removal of extra genes from BAC clones to produce BAC transgenic mouse lines. In one study a modified BAC clone was subjected to restriction digestion and PFGE, and fortuitously, a fragment could be excised that contained suitably large 5′ and 3′ flanking regions but excluded several extra genes originally present in the BAC clone (Chao et al., 2010). In another study a spontaneous deletion was identified in a single clone during the recombineering steps, and this deletion serendipitously removed the only extra gene present in the BAC clone (Belforte et al., 2010). However, the recombineering-based method we have outlined for BAC trimming affords greater reliability and precision for routine deletion of extra genes.

### Inactivation of potentially confounding “extra” genes by minimal insertion mutations

Although BAC trimming can be a versatile and powerful method for deleting both small and large segments of BAC DNA, in some cases it is not permissible to apply this method for the removal of extra genes. For example, when an extra gene is located within the critical 5′ and 3′ flanking regions of the targeted gene, deletion of these flanking regions would likely negatively affect the pattern of gene expression. Thus, we devised a recombineering strategy to inactivate the *Sfxn1* gene nested within the putative promoter region of the *Drd1a* gene by inserting stop codons in all three reading frames to interrupt the coding region of *Sfxn1*. In this manner the inactivation of an extra gene can be carried out with minimal insertions as small as ~15 bp, but in our case we have designed a 29 bp sequence (see Materials and Methods) for targeted inactivation of *Sfxn1* gene in the *Drd1a*-spanning BAC RP24-47M2 that further introduces unique restriction sites for diagnostic purposes (Figure 4E). The targeting vector for gene inactivation does not contain any selection marker and therefore must be used in conjunction with a second targeting vector that does contain a selection marker, in this case either an iTV-based targeting vector or a BAC trimming cassette.

In order to ensure that the antibiotic selection appropriately isolates double recombinant clones, the concentrations of the linearized targeting vectors were carefully proportioned. The linearized Sfxn1 Target and iTV1-D1-ChETA Target DNA were combined at ~20:1 for the dual targeting step in EL250 cells propagating RP23-47M2 in order to favor a high likelihood of isolating clones with successful inactivation of *Sfxn1*. Initial attempts did not yield correct double recombinant clones, but instead, only the ChETA cassette with selection marker were found to be inserted in recovered BAC clones. The results were markedly improved by using high quality MAXI prep DNA of high concentration, ensuring the linearization was complete with no uncut plasmid remaining, and use of LB Lennox media instead of LB Miller in the recombineering protocol. With these changes the majority of experiments yielded one or more correct double recombinant BAC clones with insertion of the ChETA expression cassette downstream of the *Drd1a* promoter region and inactivation of *Sfxn1* by insertion of the three-frame stop mutation immediately downstream of the *Sfxn1* start codon. The insertion of the inactivation cassette incorporated a new diagnostic SalI restriction site adjacent to the three-frame stop mutation, and thus, it was feasible to verify *Sfxn1* inactivation at the DNA level by SalI digestion and PFGE (Figure 4F).

### Preserved cell-type specific expression patterns in BAC transgenic mice created with advanced recombineering strategies

**Figure 5:**
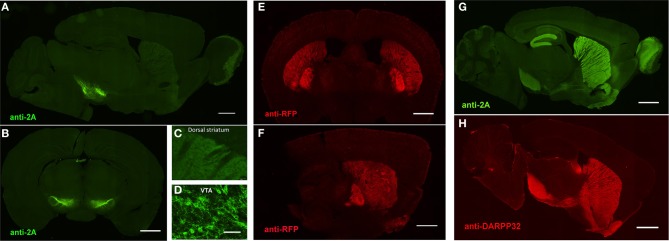
**Preserved cell-type specific expression patterns in BAC transgenic mice created with advanced recombineering strategies. (A–D)** Anti-2A slice staining in brain slices from DAT-ChETA line 3 mice reveals the distribution of membrane-targeted ChETA protein. Sagittal **(A)** and coronal **(B)** sections showing ChR2 expression in midbrain dopamine neurons. **(C,D)** High magnification images of axon terminal labeling in the dorsal striatum **(C)** and labeled neurons in the ventral tegmental area **(D)**. **(E,F)** Anti-RFP slice staining in brain slices from A2A-ChETA line 13 mice showing tdTomato expression in striatopallidal medium spiny neurons (MSNs) in coronal **(E)** and sagittal **(F)** sections. Lighter expression can also be detected in putative cortical astrocytes. **(G)** Anti-2A slice staining in a sagittal brain slice from D1-ChETA line 1 mice showing ChETA expression in striatonigral MSNs. Additional expression is apparent in other brain regions, particularly the dentate gyrus, layer VI cortex, and olfactory bulb. **(F)** Anti-DARPP32 slice staining to label both striatonigral and striatopallidal MSNs. Scale bars: 1 mm in **(A,B,E–H)** and 100 μm in **(C,D)**.

**Figure 6:**
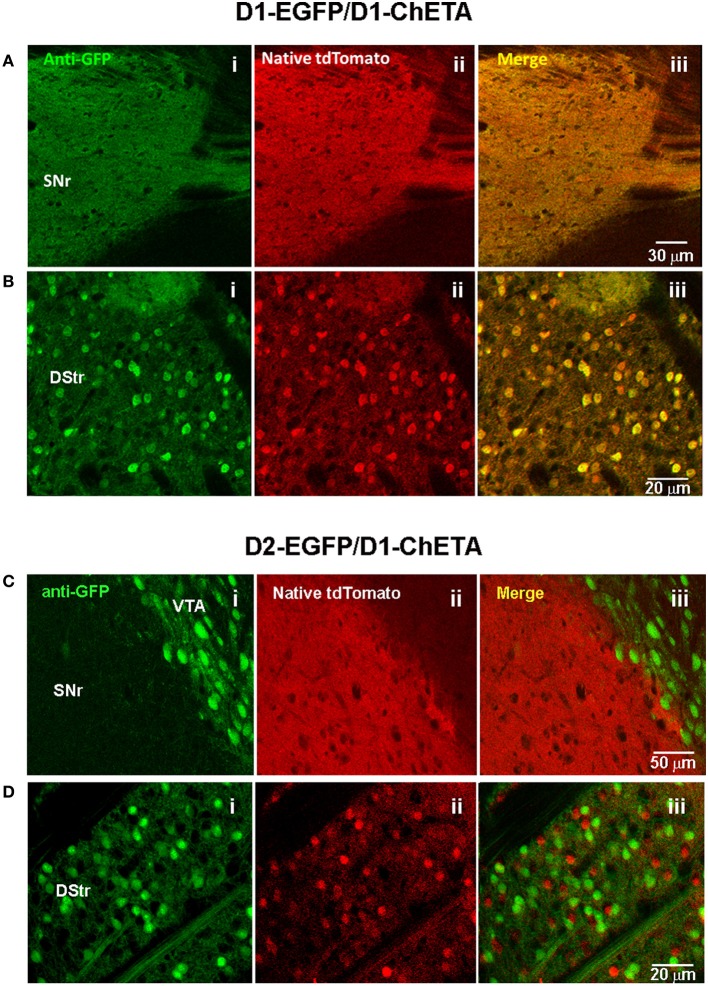
**Validation of transgene expression in striatonigral but not striatopallidal MSNs in D1-ChETA line 1 mice. (A,B)** Confocal images in brain slices from D1-EGFP/Drd1a-ChETA double transgenic mice. **(A)** Substantia nigra pars reticulata (SNr) region showing anti-EGFP **(Ai)**, native tdTomato fluorescence **(Aii)**, and the merged image **(Aiii)**. **(B)** Images from the dorsal striatum (DStr) region. **(C,D)** Confocal images in brain slices from D2-EGFP/D1-ChETA double transgenic mice. **(C)** SNr/VTA boundary showing anti-EGFP **(Ci)**, native tdTomato fluorescence **(Cii)**, and the merged image **(Ciii)**. **(D)** Images from the DStr region.

An important consideration for the BAC trimming and gene inactivation strategies employed here is whether or not the BAC DNA manipulations ultimately negatively impact the transgene expression pattern in transgenic animals. Although these strategies appear highly effective for removal of unwanted genes from BAC clones, the utility of such manipulations would be greatly diminished if the cell type-specific expression patterns were not preserved. To demonstrate faithful expression we created BAC transgenic mouse lines for expression of the fast kinetic ChR2 variant ChETA under the regulatory elements for *Adora2A* (A2A), *Drd1a* (D1), and *Slc6a3* (DAT). The BAC clones were modified as described using BAC trimming or gene inactivation strategies such that no unwanted extra genes were present. The modified BAC clones were used for pronuclear injection to create BAC transgenic founders. In each case we were able to successfully identify a single founder line for A2A-ChETA, D1-ChETA, and DAT-ChETA with functional transgene expression matching to the respective endogenous expression patterns (Figure 5). In order to further validate transgene expression in D1-ChETA mice we crossed these mice to the well characterized D1-EGFP or D2-EGFP GENSAT BAC transgenic mice. Our analysis revealed a near perfect overlap of EGFP and tdTomato (proxy for ChETA) restricted to striatonigral medium spiny neurons (MSNs) in D1-EGFP/D1-ChETA double transgenic mice (Figures 6A,B). In contrast, virtually no overlap in EGFP and tdTomato expression was observed in the dorsal striatum region of D2-EGFP/D1-ChETA double transgenic mice indicating exclusion of ChETA expression from striatopallidal MSNs (Figures 6C,D).

## Discussion

We have designed and implemented BAC recombineering strategies for eliminating undesirable extra genes from BAC clones in order to circumvent confounds due to overexpression of extra genes in BAC transgenic lines. The BAC recombineering strategies are simple, efficient, and reproducible and have been implemented to successfully modify numerous mouse BAC clones from the RP23/RP24 library. Our BAC trimming strategy enables precisely targeted deletions of undesirable BAC DNA regions, such as those stretches harboring off-target extra genes. By judicious selection of BAC clones, in most cases it is feasible to remove all unwanted extra genes with a single deletion step targeting only one end (rather than both ends) of the BAC. We have demonstrated precise deletions of up to 111 kb and spanning as many as four unique genes in a single recombineering event with no evidence for any upper size limit thus far. Notably, excessive removal of large expanses of BAC DNA can be detrimental to the intended transgene expression pattern. Thus, although BAC trimming may be optimal for a wide variety of applications, it may not be feasible to fully remove all unwanted portions of BAC DNA for every BAC clone of interest.

In some BAC clones a gene of interest may occasionally be found to reside in very close proximity to a separate off-target gene, as was the case for the *Drd1a* and *Chat* spanning BAC clones in our study. In such cases a simple BAC trimming strategy is not suitable since deletion of the unwanted extra gene would also eliminate putative 5′ or 3′ regulatory elements essential for specifying the unique expression pattern of the targeted gene. Thus, a gene inactivation strategy was implemented to insert a three-frame translational stop mutation plus diagnostic restriction site immediately downstream of the initiating methionine start codon in the unwanted extra gene. The size of the inactivation cassette is intentionally minimal (e.g., lacking antibiotic selection cassette) and serves to eliminate functional gene expression from the unwanted extra gene without additional disruption to important regulatory elements in the region. The BAC recombineering strategy for gene inactivation requires dual targeting together with a second targeting cassette incorporating a functional selection marker. This dual targeting strategy allows for simultaneous insertion of a transgene cassette and inactivation of an extra gene by selecting for clones that have undergone successful homologous recombination at both locations within the BAC DNA sequence—i.e., double recombinant clones. In order to ensure a high likelihood that the antibiotic selection appropriately isolates double recombinant clones, the concentrations of each linearized targeting vector must be carefully proportioned. With optimization, the dual homologous recombination step was efficient enough to routinely identify multiple successfully targeted clones in a single attempt.

The overall goal of our efforts was to remove all extra genes from the selected BAC clones using either or both of these versatile recombineering strategies, leaving only a single targeted gene with sufficient 5′ and 3′ flanking regions to direct faithful gene expression *in vivo* in transgenic mice derived from pronuclear injection of the modified BAC DNA. In essence, the resultant modified BACs could be considered as designer “mini BACs” that retain only the most essential elements from the parent BAC clone while removing all non-essential portions. In designing our approaches we have had excellent initial success following a general guideline of retaining at least 50 kb of 5′ sequence and 15–20 kb of 3′ sequence flanking the targeted gene within BAC clones; however, the extent of 5′ and 3′ regions required must be determined empirically on a case-by-case basis. Once a successfully modified BAC clone is validated for proper cell-type specific expression, the same BAC modification strategy or preferably the same trimmed BAC clone itself can then be re-purposed for subsequent recombineering and expression of virtually any other transgene of interest. We provide a list of several BAC clones that have been modified using these methods and that can be utilized to target transgene expression to defined neuronal subsets (Table 2).

**Table 2:** **Successfully modified BAC clones using BAC trimming and gene inactivation approaches**.

**Targeted gene**	**BAC ID**	**BAC size (kb)**	**deletion size (kb)**	**trimmed BAC size (kb)**	**deleted genes**	**inactivated genes**	**insertion size (bp)**
*Drd1a*	RP23-47M2	283.0	–	–	–	*Sfxn1^^^*	29 bp
*Drd2*	RP23-161H15	226.8	77.9	148.9	*Ttc12, Ankk1*	*–*	*–*
*Adora2a*	RP24-238K3	186.0	50.8	135.2	1110038D17Rik-210^*^, Upb1	*–*	*–*
*Slc6a3 (DAT)*	RP24-269I17	199.7	84.0	115.7	Clptm1l, Tert, Slc6a18, Slc6a19^*^	*–*	*–*
*Pvalb*	RP24-306A6	140.4	52.4	88.0	*Ift27*	*–*	*–*
*Viaat (VGAT*)	RP24-246L1	177.0	111.0	66.0	Actr5, Ppp1R16b^*^	*–*	*–*
*Chat*	RP24-256F2	176.2	43.5	132.7	1700024G13Rik, Ercc6^*^	*Slc18a3^^^*	29 bp

* *Partial gene sequence only*.

^ *Gene inactivation by insertion of a 3-frame triple STOP cassette*.

The approaches we have described for modifying BAC DNA using recombineering in *E. coli* build upon and extend the work of others. In three earlier studies BAC trimming (sometimes also called BAC shaving) was similarly accomplished by targeted replacement of a DNA region with a selection marker using homologous recombination (Hill et al., 2000; Testa et al., 2003, 2004). The end goal of such BAC DNA manipulation strategies was either to create precise vectors for traditional gene targeting in mouse embryonic stem cells or to reduce redundant effort in genome sequencing projects. In contrast, we applied BAC trimming to eliminate extra off-target genes so as to avoid potentially confounding effects in mice derived from pronuclear BAC DNA injections.

A substantially different method of retrieving mini BAC fragments was previously described. This method utilized PCR and gap repair in *E. coli* to subclone ≤80 kb BAC fragments into the low copy number pBR322 vector (Lee et al., 2001). However, the resulting transgene expression patterns were highly variable and did not fully recapitulate the endogenous gene expression patterns. This stands in contrast to our results demonstrating excellent preservation of the intended cell-type specific transgene expression patterns in transgenic mice derived from BAC DNA modified using our improved strategies. This difference is likely due to the strict size limitation for sub-cloning DNA fragments into pBR322, whereas no practical size limitation is expected with typical BAC vectors such as pBACe3.6 and pTARBAC1, particularly since BAC DNA is being trimmed rather than retrieved by gap repair. Thus, the approaches that we have described seem to be more flexible when selection of considerably larger 5′ or 3′ regions surrounding the target expression cassette are required for achieving faithful expression *in vivo*, as is most often the case.

There are now extensive repositories of BAC transgenic mouse lines available, including over a thousand distinct Cre driver and EGFP reporter lines produced by the GENSAT project alone. We analyzed the full collection of BAC clones used for creating the extensive GENSAT Cre driver repository as a representative data set and determined that 75% of the BAC clones contain at least one or more extra genes. Even more surprisingly, one-third of the BAC clones contained at least three or more extra genes and 5% contained at least nine or more extra genes. The most extreme case was a BAC clone that spanned fourteen different fully intact extra genes, and this clone was used to create both Cre driver and EGFP reporter lines (*Thbs3*-spanning BAC clone RP23-368D24). Despite the extensive proven utility of numerous GENSAT lines, it is an unfortunate possibility that many available BAC transgenic lines may suffer from confounds of varying degrees due in part to overexpression of one or more “extra” genes. In some cases the unintended overexpression of one or more extra genes in various tissues of transgenic animals may have profound consequences and implications for interpreting experimental results. In other cases the extra genes may not be expressed at functionally relevant levels or perhaps there may be no measurable impact despite high level expression (Heiman et al., 2008). In support of the former, recent experimental evidence has emerged claiming significant behavioral and functional alterations in several commonly used BAC transgenic mouse lines (Ade et al., 2011; Bagetta et al., 2011; Kramer et al., 2011; Kolisnyk et al., 2013), thereby raising concerns about some aspects of the findings from the numerous studies that have implemented these lines to date. Although some aspects of the reported defects have been disputed or reconciled (Chan et al., 2012; Nelson et al., 2012), this controversy highlights the need for more rigorous systematic characterization of BAC transgenic lines. In the study by Kolisnyk et al. (2013) it was clearly demonstrated that the measured behavioral and physiological alterations were the direct result of high BAC copy number insertion and unintended overexpression of VAChT in *Chat-ChR2(H134R)-EYFP* line 6 BAC transgenic animals (Kolisnyk et al., 2013). Given this finding, further detailed investigations are likely to reveal significant impacts of overexpressed extra genes in many other widely used BAC transgenic lines. This point underscores the potential benefits of the improved BAC recombineering strategies we have developed for removing or inactivating extra genes for the purpose of creating transgenic tools with fewer potential confounds.

Beyond the limited examples we have cited above, there are undoubtedly many commonly used BAC transgenic lines for which behavioral or physiological confounds are likely but have yet to be directly assessed. As an example, several BAC transgenic lines have been reported for transgene expression in cholinergic neurons using identical or highly similar *Chat*-spanning BAC clones as the one used in developing the *Chat-ChR2(H134R)-EYFP* line 6 mice. These include a *Chat-*Cre driver line (Gong et al., 2007), two distinct *Chat-EGFP* reporter lines (Gong et al., 2003; Tallini et al., 2006), and a *Chat-L10a-EGFP* BacTRAP line (Doyle et al., 2008). Furthermore, a *Chat-*Cre BAC transgenic rat was developed by pronuclear injection of the identical *Chat-*Cre mouse BAC clone used to create the *Chat-*Cre mouse line (Witten et al., 2011). Thus, although no direct assessment of VAChT overexpression has been reported for these five independent lines, it is expected that all will have some degree of overexpression of VAChT (and potentially *Ogdhl* and *1700024G13Rik*) depending on the BAC transgene copy number for each line. However, it is worth noting that high BAC copy number insertions as reported for the *ChAT-ChR2(H134R)-EYFP* line 6 tend to be far less common as opposed to low BAC copy number insertions.

In the long-term, we propose that the most effective strategy will be to re-engineer and develop new BAC transgenic lines using improved BAC recombineering strategies to eliminate non-essential portions of BAC DNA, including (but not limited to), methods described in this study, and to deposit validated mini BACs into an open source repository for widespread use. The versatile new resources we have developed (e.g., BAC modification vectors and successfully modified BAC clones) and the simple and efficient methods we have demonstrated should facilitate progress in this area by providing further options to support diverse recombineering needs. Furthermore, with advances in pronuclear injection-based targeted transgenesis (Ohtsuka et al., 2010; Tasic et al., 2011) and identification of “safe” genomic docking loci permissible for high level expression, it may soon be possible to develop BAC transgenic animals that are free of both confounding extra genes and detrimental positional effects from random transgene integration. Continued technological innovation will help to ensure an ever-expanding collection of highly useful BAC transgenic tools that can pave the way to deciphering the functions of the remarkably diverse cell types in the brain.

### Conflict of interest statement

The authors declare that the research was conducted in the absence of any commercial or financial relationships that could be construed as a potential conflict of interest.
